# Camera trap placement and the potential for bias due to trails and other features

**DOI:** 10.1371/journal.pone.0186679

**Published:** 2017-10-18

**Authors:** Joseph M. Kolowski, Tavis D. Forrester

**Affiliations:** Smithsonian Conservation Biology Institute, National Zoological Park, Front Royal, Virginia, United States of America; University of Bern, SWITZERLAND

## Abstract

Camera trapping has become an increasingly widespread tool for wildlife ecologists, with large numbers of studies relying on photo capture rates or presence/absence information. It is increasingly clear that camera placement can directly impact this kind of data, yet these biases are poorly understood. We used a paired camera design to investigate the effect of small-scale habitat features on species richness estimates, and capture rate and detection probability of several mammal species in the Shenandoah Valley of Virginia, USA. Cameras were deployed at either log features or on game trails with a paired camera at a nearby random location. Overall capture rates were significantly higher at trail and log cameras compared to their paired random cameras, and some species showed capture rates as much as 9.7 times greater at feature-based cameras. We recorded more species at both log (17) and trail features (15) than at their paired control cameras (13 and 12 species, respectively), yet richness estimates were indistinguishable after 659 and 385 camera nights of survey effort, respectively. We detected significant increases (ranging from 11–33%) in detection probability for five species resulting from the presence of game trails. For six species detection probability was also influenced by the presence of a log feature. This bias was most pronounced for the three rodents investigated, where in all cases detection probability was substantially higher (24.9–38.2%) at log cameras. Our results indicate that small-scale factors, including the presence of game trails and other features, can have significant impacts on species detection when camera traps are employed. Significant biases may result if the presence and quality of these features are not documented and either incorporated into analytical procedures, or controlled for in study design.

## Introduction

Camera trapping has become an increasingly widespread and useful tool for wildlife ecologists, behavioral ecologists, and wildlife managers [1, 2]. One of the most common applications of this tool has been the use of capture-recapture methods to estimate the density of species with individually unique markings (e.g. tiger: [3]; jaguar: [4], ocelot: [5]). However, the vast majority of species in the terrestrial mammal community do not have unique markings, and there is great interest in the potential to answer meaningful research and management questions using camera trap photos of these species [6–9]. In an applied setting, estimates of the system state (e.g. represented by abundance, occupancy or species richness) are often the most critical for informing management decisions. In these scenarios where animals cannot be individually identified, inference about the system state must rely on either photo capture rate (as a potential index of abundance), or presence/absence information (e.g. for estimation of occupancy or species richness). A common study design approach is to distribute camera locations randomly or systematically (e.g. regular grid over study area), often at a predetermined trap density, then select an “optimal” camera placement at or near these planned locations, often targeting roads, game trails or other concentrations of animal sign [9]. However, it is increasingly clear that these camera placement strategies can have significant consequences for the reliability and applicability of resulting photo-based information [10], and best practice suggestions on where to place cameras to minimize bias in these cases are poorly developed [9].

The rate of photo captures as well as the probability of obtaining a photograph of a given species at a given location are a function of two processes, the abundance of a species in a given location, and its likelihood of being photographed at that location (detection probability). While it is clear that a range of biological and behavioral factors like species size [11, 12], home range size [10], and social rank [13] influence detectability in camera traps, the relationship between detectability and local habitat features is less clear. Yet mounting research indicates that camera placement decisions are a critical consideration and are a potentially large source of bias in detection rates. Large and medium-sized carnivores have been shown to prefer using roads and human trails as travel routes [10, 14–17], and cameras placed on these features resulted in higher capture rates of these species. There is evidence that prey species also show varying levels of attraction to or avoidance of these larger travel routes [15, 18, 19], and that these biases may vary based on trail/road width and age [20]. Relative attraction or avoidance may not be consistent even within some species ([19, 20]—tapirs and peccaries), further complicating the analysis of photo data across studies.

Given what is known about these biases, any study attempting to make robust, unbiased conclusions based on capture rate or presence/absence data have essentially two options. The first is to avoid selection of “optimal” camera placements entirely. If camera placements are truly random in the landscape, then features influencing detection will be included in proportion to their availability. While this does not remove detection biases inherent across or within species (e.g. size, movement rate, range size, social rank), it is a more statistically defensible sampling strategy, with truly random sampling allowing inference beyond the specific locations where cameras are placed. For this reason random camera placement (with respect to landscape features) is an assumption of a relatively new approach that aims to estimate density from non-individually identifiable species [7]. There is however a reluctance among researchers to use completely random placement due to anticipated reductions in overall photo capture rate (demonstrated in [10, 21] and others) and so it is remarkably rare in practice [9]. Recently some authors have suggested a mix of trail and off-trail (“random”) placements for studies seeking a compromise between bias and reduced capture rate [15, 21, 22]. Yet this stratification of placement decisions will have unknown consequences on overall detection rates and will influence capture rates of different species in different ways. Further, it assumes that trail/road features are the only significant habitat feature influencing detection rates.

The second option is to account for detection parameters analytically, regardless of placement strategy. This approach has been well developed in an occupancy modeling framework [23]. Occupancy models, which often seek to describe the factors influencing patch (site-level) occupancy or use of a species, separate the process of detection from presence, allowing researchers to explicitly model factors that influence detection. In camera studies, individual or blocks of days are considered repeated sampling events at a given site, and allow direct estimation of detectability. Use of this approach has allowed researchers to investigate ecological questions using camera traps while explicitly accounting for many of the biases inherent in photo capture rate (e.g., [24–27]). While this approach is largely restricted to presence/absence data, model expansions allow additional information related to abundance to be utilized [28]. Importantly, this approach still relies on accurate knowledge about the factors that can influence detection probability so they can be recorded in the field and included in modeling efforts.

Although we continue to understand more and more about the potential biases involved in camera placement, most studies on this topic to date have focused on the new world tropics, and on the influence of “roads” of various sizes (i.e. travel routes of human origin). Much less is known about detection bias along smaller travel paths (e.g. game trails), which are frequently targeted for camera placement in a range of scenarios, or how these various biases may change under different habitat conditions. It has been suggested that travel routes and other features may be more attractive to various species in thicker vegetation (e.g. [21]), but this has not been adequately investigated, and the suggestion was generally not supported in the single study where this pattern was investigated [15]. Finally, we know nothing about the potential influence of other small scale habitat features on animal detection.

Given that estimates of system state parameters of interest such as occupancy probability and species richness are heavily reliant on the detection process, our objective in this study was to clarify the extent to which the detectability of species may vary based on small-scale habitat features at camera locations. We focused on two factors of probable importance in our study system, game trails (no human use) and downed logs. We hypothesized that game trails would be used preferentially for travel and that downed logs would be used for foraging or travel by several species [29]. We utilized paired cameras, to date rarely employed on a large scale for this question (but see [30]), to control for any larger-scale confounding factors (e.g. site occupancy, site use, animal abundance) that may influence detectability of species presence at a location. We also recorded a suite of vegetation-based habitat variables in the immediate vicinity of camera pairs to account for their influence on local species occupancy, and to investigate the extent to which local habitat structure may also influence detection rates. Our study was based in the broadleaf deciduous forests of the Shenandoah Valley, Virginia, USA, a system where relatively little camera trap methodology research has been conducted (but see [31]). We investigated the influence of random vs. feature-based camera placement on estimates of species richness and the probability of detection of a suite of species in this forest mammal community.

## Materials and methods

### Study area

Our study was conducted in forested habitats of varying ages on the grounds of the Smithsonian Conservation Biology Institute (SCBI) in Front Royal, Virginia, USA (38.887090° N, 78.164652° W). The 1295 ha campus is near the border of the Shenandoah National Park. Forest canopies are dominated by Tulip Poplar (*Liriodendron tulipifera*), Ash (*Fraxinus spp*.) and a suite of Oak (*Quercus spp*.) and Hickory (*Carya spp*.) species. Forest understories varied from very open, to those with relatively dense growths of Coralberry (*Symphoricarpos orbiculatus*), Japanese Barberry (*Berberis thunbergii)*, and various members of the *Rubus* genus, as well as the occasional monoculture understory of Spicebush (*Lindera benzoin*). Forests of varying ages cover approximately 66% of the entire property. The most common terrestrial large mammal is the white-tailed deer (*Odocoileus virginianus*), and black bear (*Ursus americanus*) are more abundant here than in other areas of their North American range. Common large rodents include eastern gray squirrels (*Sciurus carolinensis*), eastern fox squirrels (*Sciurus niger*), and the eastern chipmunk (*Tamias striatus*). Other abundant small and mid-sized mammals are the Virginia opossum (*Didelphis virginiana*), northern raccoon (*Procyon lotor*), and eastern cottontail rabbit (*Sylvilagus floridanus*). The predator community also includes bobcats (*Lynx rufus*), gray fox (*Urocyon cinereoargenteus*), red fox (*Vulpes vulpes*), and coyote (*Canis latrans*). Approximately 3.3% of the property is allocated for care and breeding of captive non-domesticated species that reside in large barns and fenced enclosures, and an additional 11.3% is available as fenced pasture for the captive animals at various times. Captive animals do not interact with native mammals, and all captive ungulates are kept within a larger fenced area that is kept free of all native deer. The property is contiguous with the nearly 81,000 ha forest of Shenandoah National Park, and although the property is fenced, the perimeter fences are permeable to all local wildlife species. The climate is classified as humid subtropical, with hot humid summers and mild cool winters. Snowfall is irregular but common after January.

### Study design

The entire SCBI property was overlaid with a systematic grid (500m x 500m) with a random starting point, and with grid center points designated as target locations for a paired camera station. All points that fell within the central developed campus or the captive animal areas were removed from consideration. No cameras were located less than 100m from any animal enclosure. Center points that were > 200m from forest habitat were also removed from consideration, either based on satellite images or after traveling to the location, leaving 42 potential station locations. We traveled to these remaining locations in random order and searched for either of our feature types (game trail or log) occurring in forested habitat. Although a minimum log diameter threshold was not designated, no logs below a diameter of 19.5 cm were selected. Log length was assumed to correlate closely with diameter and so was not recorded, yet all logs were significant habitat features and were at least 3m long. We considered a game trail for inclusion in the study if signs of animal movement appeared to be repeated as opposed to single use, and if the ground was at least partially bare on the trail due to use by wildlife. Old roads and fencelines were never used as trail features and all cameras were restricted to be at least 50m from an actively used road or human use trail.

All camera sets were established as pairs, with one camera (“feature camera”) focused on the microhabitat feature of interest, and the other (“control camera”) placed as close as possible to the first camera (goal of within 15m), yet not including the feature of interest. We used the paired design to control for detection biases inherent in our different camera models, as well as to remove the influence of the landscape location and surrounding habitat on species presence between paired cameras. That is, paired cameras were close enough to assume that overall site occupancy for any species was equivalent at both cameras. For trail feature pairs, the goal was to have the two cameras as close as possible but with the control camera not in direct sight of the trail to avoid animals leaving the trail to investigate the control camera. We placed control cameras such that animals using the “feature” would never set off the control camera. In all pairs, we ensured that even if the distance between cameras exceeded our goal of <15m, the general microhabitat features surrounding the feature camera were the same at the control camera, particularly with respect to the density and type of understory vegetation, and the ground cover characteristics. We collected data for this study during the months of June through December (though only four camera deployments extended into December) in both 2013 and 2014. Our goal was to have camera pairs in position for a minimum of three weeks before being moved to a different grid location or feature type; enough time to at least have a non-zero probability of capture for all photographable mammal species in this ecosystem [29].

Two camera brands were used in the study: SpyPoint (BF-7; Swanton, Vermont, USA) and Reconyx (RC-60 Covert IR and HyperFire HO Covert IR, Holmen, Wisconsin, USA). All three models employ a low-glow infrared flash and in all cases pairs were composed of only one camera model. Reconyx cameras comprised 37.5% of the trail feature deployments and 43.3% of log feature deployments. All cameras were set to maximize the number of photos taken. Reconyx cameras were set to Rapidfire (no delay between photos) setting, three pictures per trigger, maximum sensitivity, with no quiet period and had a 1/5 second trigger speed. Spypoint cameras were set to take 4 photos at each trigger (maximum setting) at maximum sensitivity distance, with no delay between photos and 1 minute between triggers (minimum setting), with an estimated 1.5 second trigger speed. To maximize detection of the largest range of animal sizes, cameras were set at or below knee height (~ 40cm), except when modified heights were required to photograph animals traveling along the log in the camera view at log experiment stations. Generally we attempted to maximize the detection distance of the cameras at each placement (using each camera’s test mode) while minimizing camera height.

At each paired camera station we collected a suite of measurements representing vegetation and habitat structure to investigate the influence of local habitat structure on detection probability and to account for its potential influence on probability of local occupancy (Table 1). The midway point between the two cameras was used as the center of vegetation surveys. At each station we also recorded log diameter (cm; at the center of the view of the camera), trail quality (average, good, excellent, Table 1) and the distance between the control and feature cameras. Because sampling extended from summer into fall, each deployment was categorized by season based on the mid-date of the session (past Oct 15 = Fall).

**Table 1:** Covariates used in multi-method occupancy modeling of camera trap detections.

Covariate	Modeled on	Collection Protocol
Overall vegetative cover (CovAll)	*θ*	Average percent of a 2-m high cover pole (located at midway point between the two cameras) obscured by vegetation from 10 m away in each cardinal direction. Coverpole was divided in 20 1-dm sections. Data were recorded as the percentage of 1-dm sections obscured (>50%) by vegetative or structural cover.
Low vegetative cover (CovLow)	*θ*, *p*	Average percent of lower portion (<0.5 m) of cover pole obscured by vegetation
Understory stem density (UndStD)	*θ*, *p*	Number of woody stems >1.5 m in height and <7.5 cm dbh counted on three transects. Transect were parallel, 20-m transects extending perpendicular to the aspect of the location. Transects were placed both through the midway point between the two cameras, as well as 10 m up and down slope of this location. Transect width was determined by the extent of the surveyor’s outstretched arms.
Overstory stem density (OverStD)	*θ*	Number of woody stems >7.5 cm dbh counted on transects (as described for UndStD)
Camera Type (CamType)	*p*	Camera used at each paired station. Reconyx (coded as “1”) and Spypoint (coded as “0”).
Season	*p*	Dominant season of each sampling session, based on the mid-date of the full session. Mid-dates past October 15th, were coded as Fall (“0”) as opposed to Summer (“1”).
Log Diameter (LogD)	*p*	Diameter of the primary log in view of the camera, in centimeters, measured in the center of the camera’s view.
Trail Quality (TrailQ)	*p*	Subjective assessment of trail quality designed to represent amount of trail use by wildlife. Ranged from average (“3”) to good (“2”) to excellent (“1”) based on presence of animal tracks, amount of bare ground exposed, and trail width.

Vegetation measurements were centered on the midway point between the two cameras at each station. The *θ* parameter in the modeled column represents local occupancy, or the probability that a species is present in the immediate vicinity of the cameras. The parameter *p* represents detection probability.

### Data analysis

To compare the rate at which new species were accumulated using the various tested camera placements, we generated sample-based rarefaction/extrapolation curves for species richness for feature and control cameras of our log and trail experiments and compared them using 95% confidence intervals drawn from 500 randomizations performed with replacement and based on the unconditional variance [32]. In addition, we calculated species richness for each feature and control camera and compared average richness values within groups using a Wilcoxon signed-rank test.

Photo series at the same camera for the same species were considered independent if 10 minutes passed with no captures of the respective species. Photo capture rates, both within and across species, were calculated as: (number of independent events divided by the number of camera nights) multiplied by 100. Comparisons of capture rates were performed using a Wilcoxon signed-rank test, treating each camera at each deployment as a sample. Survey effort was always controlled within pairs, such that data was only utilized as long as both cameras were functioning; any data recorded after one camera stopped functioning was ignored.

We modeled detection probability within a multi-method occupancy modeling framework [33]. This modeling framework is uniquely suited to investigate the effect of method (in this case, camera placement) on detection in a paired design, as it incorporates a local occupancy parameter (*θ*) in addition to the traditional site occupancy parameter (*ψ*). In this model local occupancy (*θ*) describes the probability that the region in the immediate vicinity of the two cameras is occupied, while the site occupancy (*ψ*) parameter represents the probability that the broader sampling location, here defined by our grid cells, is occupied. With this additional *θ* parameter these models explicitly address the lack of independence among detections by the two paired cameras at the same sampling occasion induced by animal(s) presence in the immediate vicinity of the cameras. This multi-method modeling approach also effectively utilizes the data inherent in the two camera setups to estimate both occupancy and method-specific detection, as opposed to treating the two cameras as separate, independent sites. This approach allows direct estimation and modeling of occupancy parameters at a local and sampling grid scale. In our case, all of the modeled species are known to be present throughout our study area, so the *ψ* parameter was not of interest in this context. In addition, the paired design controls for any potential station to station variation in species presence and abundance. However the addition of the *θ* parameter allows a framework where the probability that a species is locally available for photographic capture, which is of much more interest in this study, is directly estimated and taken into account. This approach also allows for testing of method-specific hypotheses in a model selection framework.

Detection histories were created for each camera and each species, with each sampling occasion representing two days. To minimize the amount of missing data in the detection matrix caused by some cameras that remained in deployment for longer periods, we excluded data collected after day 24, resulting in 12 occasions. In the multi-method occupancy framework [33], three parameters are estimated: standard site occupancy (*ψ*), detection probability (*p*), which can be estimated separately for each method, and the additional parameter of local occupancy (*θ*), which represents the probability that the species is in the immediate area of the traps. In our case, we treated our trail experiment and log experiment as separate analyses, with the feature (log or trail) and control camera placements representing our two “methods” of capture. As site occupancy was not a focus of this study, occupancy was held constant for all models. Local occupancy (*θ*) was modeled with four potential covariates (Table 1; Overall Vegetative Cover, Low Vegetative Cover, Understory Stem Density and Overstory Stem Density). Detection probability was modeled with the following suite of predictor variables: Camera Type; Season; Understory Stem Density; Low Vegetative Cover, and either Trail Quality or Log Diameter (Table 1). The latter two covariates were always modeled as an interaction effect with placement, given that these covariates would presumably have varying effects on control and feature cameras. We note that there was collinearity between Low Vegetative Cover and Overall Vegetative Cover (Pearson’s r = 0.66, p <0.05) and between Overall Vegetative Cover and Understory Stem Density (Pearson’s r = 0.76, p <0.05). However these variables were selected *a priori* to represent specific habitat components that we suspected may be important in different ways to different species. Given that no step-wise selection methods were employed, and all model combinations were investigated, collinearity was not a serious concern and we elected to maintain all our pre-selected variables in our analyses.

Model comparisons were performed separately for log and trail experiments and for each species. Model creation proceeded as follows, with the site occupancy parameter (*ψ*) held constant in all cases: 1) All possible subsets of our four covariates on *θ* (including a constant *θ*) were tested, using a global model for *p* including all covariates, and the best model using AICc (Akaike’s Information Criterion corrected for small sample sizes) values was selected. 2) With the best *θ* model, we investigated the importance of camera placement on detection probability by comparing our global model for *p* (CamType, Season, CovLow, UndStD, Placement) to the same model with *p* held constant across the two camera placements (feature and control). This comparison allowed us to clearly address whether detection probability was influenced by the trail or log placement relative to control cameras. Note that TrailQ and LogD were ignored here as they can only be included in models with placement-specific *p* estimates; 3) To investigate the relative importance of other covariates on *p*, we proceeded to test all subsets of our five covariates (CamType, Season, CovLow, UndStD, TrailQ or LogD), using the best model for *θ*, and holding *p* constant across placement methods only if detection probability was not found to be influenced by camera placement in #2. The cumulative AICc weights (given that the model sets were balanced) were used to compare relative variable importance. These are calculated by summing the individual model weights for all models that include the variable of interest, and higher cumulative weights indicate stronger influence on model fit. Although there is no generally agreed-upon threshold for cumulative AICc weight values that would indicate a variable had a “strong” or “important” impact on the response variable, we focus our discussion on those variables with cumulative AICc weights above 0.60. AICc values used an effective sample size represented by the number of successful paired deployments.

Occupancy models were generated using the software package Presence [34]. All other analyses were performed in R version 3.2.4 [35]. Species accumulation curves were generated and plotted (with some modification for visualization purposes) using the package iNEXT [36]. Detection histories were generated using the package camtrapR [37].

## Results

We successfully recorded data from a total of 54 pairs of cameras at 27 different grids in the study area (30 log sets at 23 different grids, 24 trail sets at 21 different grids). A number of deployments were cut short due to damage from black bears (*U*. *americanus*), yet in some cases both cameras functioned for multiple weeks. We decided to include all stations where both cameras functioned for at least 10 camera-nights, and in all cases we removed photos taken from both paired cameras after the date when one camera was damaged. Only 14 of the 54 sets included data from less than 21 camera-nights. The average number of camera-nights for a camera pair across the study was 25 (range: 11–50) and the study accumulated a total of 1371 camera-nights, per pair, across two sampling years (total camera nights by camera = 2742).

The average log diameter at the 30 log feature cameras was 37.2 cm (range: 19–190 cm). The log at 190 cm was an outlier and with it removed the average diameter was 32 cm. At log experiment stations, cameras were on average 15.1 m apart (range 0–35 m) and ran for an average of 25.4 camera-nights for a total of 792 paired camera nights. At trail experiment stations, cameras were on average 23.5 m apart (range 2.6 m-95.3 m) and ran for an average of 25.6 camera-nights for a total of 579 paired camera nights. The station with cameras spread over 95.3 m was an outlier and resulted from the highly linear and dense nature of the forest patch. The next largest camera distance for trail experiment stations was 53 m.

Across all stations we recorded photos of 19 different species of mammals, including the domestic cat (*Felis catus*; Table 2). Overall capture rates (often referred to as a relative abundance index) were significantly higher at trail and log cameras compared to their corresponding paired random cameras (Table 2). Across species for which there was at least 20 detections across random and feature setups, three of eight species showed significant capture rate differences in the log experiment (*O*. *virginianus*, *S*. *carolinensis*, and *S*. *niger*), and two of seven in the trail experiment (*O*. *virginianus*, *D*. *virginiana*; Table 2). Across these five significantly differing pairings, changes in capture rate ranged from 1.7 to 9.67 times. The most dramatic change in capture rate was seen for the combined “mouse” category (most likely all *Peromyscus* sp.), where 65 capture events occurred at log features compared to only a single capture event at random locations, although statistical comparison was not possible.

**Table 2:** Mean (not overall) capture rates (CR) and events (Evts.) for all 19 species photographed across 54 paired camera stations.

Species	All Cameras (n = 54 pairs)	Logs (n = 30 pairs)					Trails (n = 24 pairs)				
		Feature		Control			Feature		Control		
	# (%) of 108 cameras	CR (SE)	Evts.	CR (SE)	Evts.	Total Evts.	CR (SE)	Evts.	CR (SE)	Evts.	Total Evts.
All Species	-	**157.9 (19.6)**	1028	**111.2 (11.1)**	871	1899	**161.5 (22.3)**	863	**88.2 (13.8)**	464	1327
*Odocoileus virginianus*	105 (97.2)	**30.44 (8.77)**	224	**53.51 (9.80)**	442	666	**60.76 (8.63)**	340	**35.64 (5.25)**	196	536
*Procyon lotor*	96 (88.9)	29.45 (5.06)	205	24.63 (5.48)	190	395	27.42 (5.69	143	22.28 (4.74)	126	269
*Ursus americanus*	72 (66.7)	8.20 (1.70)	58	6.95 (1.81)	52	110	9.31 (2.13)	51	7.72 (1.86)	37	88
*Sciurus carolinensis*	69 (63.9)	**47.51 (10.50)**	318	**14.00 (4.66)**	113	431	35.18 (13.08)	191	16.10 (7.25)	74	265
*Didelphis virginiana*	43 (39.8)	3.35 (1.88)	21	4.49 (1.27)	31	52	**11.55 (4.16)**	60	**2.57 (0.83)**	15	75
All Mouse	13 (12.0)	9.36 (5.88)	65	0.15 (0.15)	1	66	2.48	14	0.60	3	17
*Urocyon cinereoargenteus*	15 (13.9)	2.51 (1.26)	19	2.51 (1.03)	20	39	0.00	0	0.17	1	1
*Sciurus niger*	15 (13.9)	**8.63 (3.61)**	78	**1.12 (0.66)**	8	86	0.22	1	0.36	2	3
*Vulpes vulpes*	14 (13.0)	0.98	8	0.45	4	12	0.94	5	0.00	0	5
*Sylvilagus floridanus*	13 (12.0)	0.16	1	0.22	2	3	3.61 (1.44)	21	0.74 (0.43)	6	27
*Canis latrans*	10 (9.3)	0.41	3	0.54	4	7	0.34	2	0.20	2	4
*Lynx rufus*	10 (9.3)	0.44	3	0.40	3	6	0.80	5	0.17	1	6
*Tamias striatus*	9 (8.3)	1.26	8	0.08	1	9	4.79 (3.95)	24	0.20 (0.20)	1	25
*Felis catus*	3 (2.8)	0.24	2	0.29	2	4	0.00	0	0.00	0	0
*Mustela frenata*	3 (2.8)	0.39	5	0.00	0	5	0.44	3	0.00	0	3
*Marmota monax*	2 (1.9)	0.22	1	0.00	0	1	0.44	2	0.00	0	2
*Mustela nivalis*	1 (0.9)	0.14	1	0.00	0	1	0.00	0	0.00	0	0
*Glaucomys volans*	1 (0.9)	1.08	10	0.00	0	10	0.00	0	0.00	0	0
*Mephitis mephitis*	1 (0.9)	0.00	0	0.00	0	0	0.11	1	0.00	0	1

Standard errors are shown when more than 20 events were recorded across cameras in each experiment. In these cases, Wilcoxon Rank Sum tests were performed and significant differences (*p* < 0.05) are in bold.

### Species richness

Excluding the domestic cat events, a total of 17 species were recorded at cameras with log features compared to a total of 13 species at their paired control cameras. Mean counts of species were higher at log features (5.17) compared with paired control cameras (4.27; V = 73.5, *p* = 0.03). Four species were photographed at log features that were never photographed at their paired random locations (long-tailed weasel—*Mustela frenata*, least weasel—*Mustela nivalis*, southern flying squirrel—*Glaucomys volans*, groundhog—*Marmota monax*) over the 792 paired camera nights. Two of these species were not photographed at any other camera placement type (*M*. *nivalis*, *G*. *volans*) over the 2742 overall camera nights (when each individual camera is considered). A total of 15 species were recorded at cameras on trails, compared to 12 species at their paired control cameras. Mean counts of species were higher on trails (5.00) compared with paired control cameras (3.71; V = 26, *p* = 0.005). Four species were photographed at game trails that were never photographed at corresponding control cameras (*M*. *frenata*, *M*. *monax*, striped skunk—*Mephitis mephitis*, *V*. *vulpes*) after 579 paired camera nights of effort. Only one species (*U*. *cinereoargenteus*) was photographed at trail control cameras and not at the trail cameras themselves.

**Fig 1:**
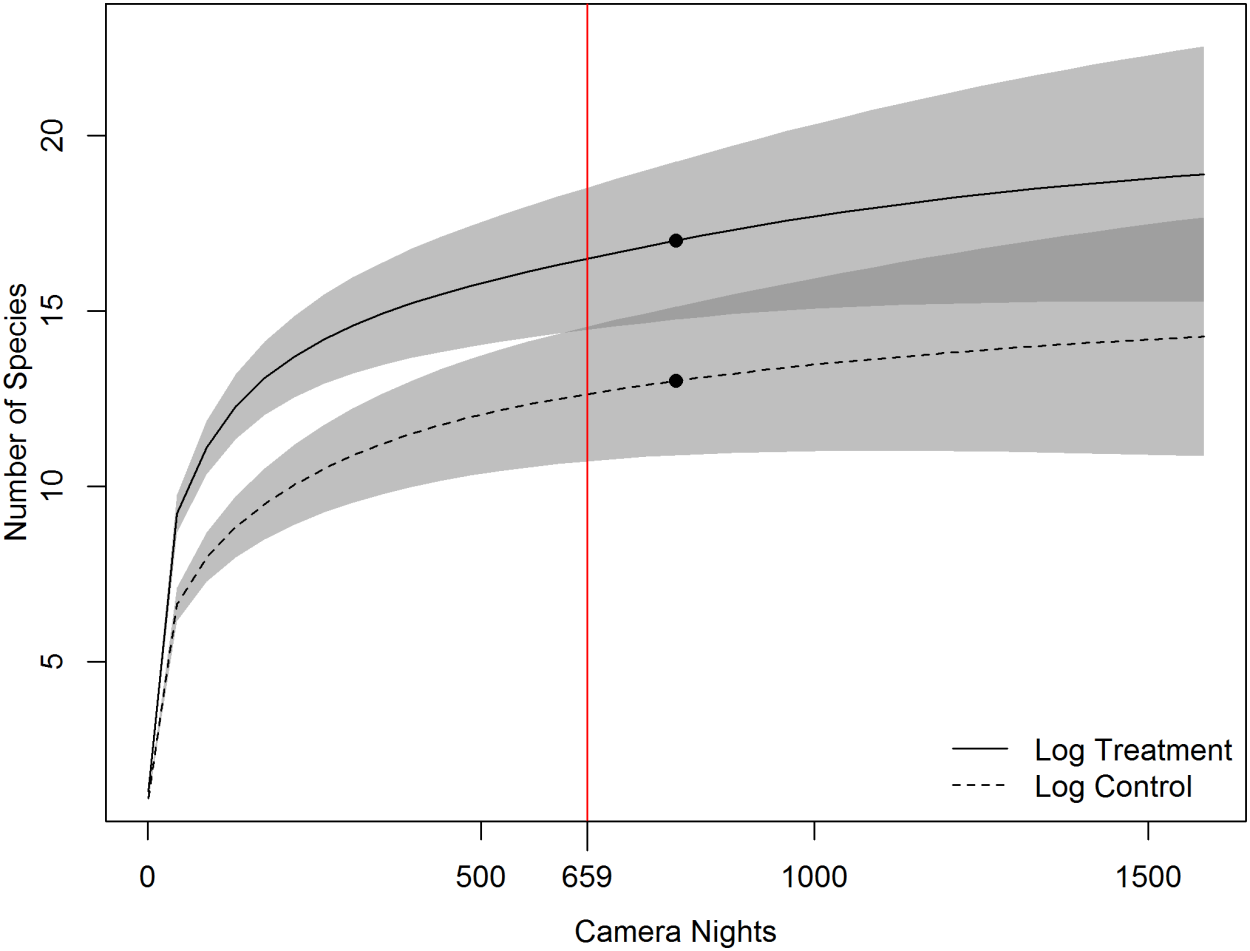
Sample-based species accumulation curve from 30 paired cameras, run for an average of 25.4 camera nights, with one camera in each pair oriented toward a log feature, and the other at a nearby (mean = 15.1 m) random location. Shaded areas represent the 95% confidence intervals, with darker shaded areas representing confidence interval overlap between the two scenarios. Black dots indicate the actual sampling effort of this study. The vertical line with label indicates the point at which the confidence intervals of the feature and control groups overlap.

**Fig 2:**
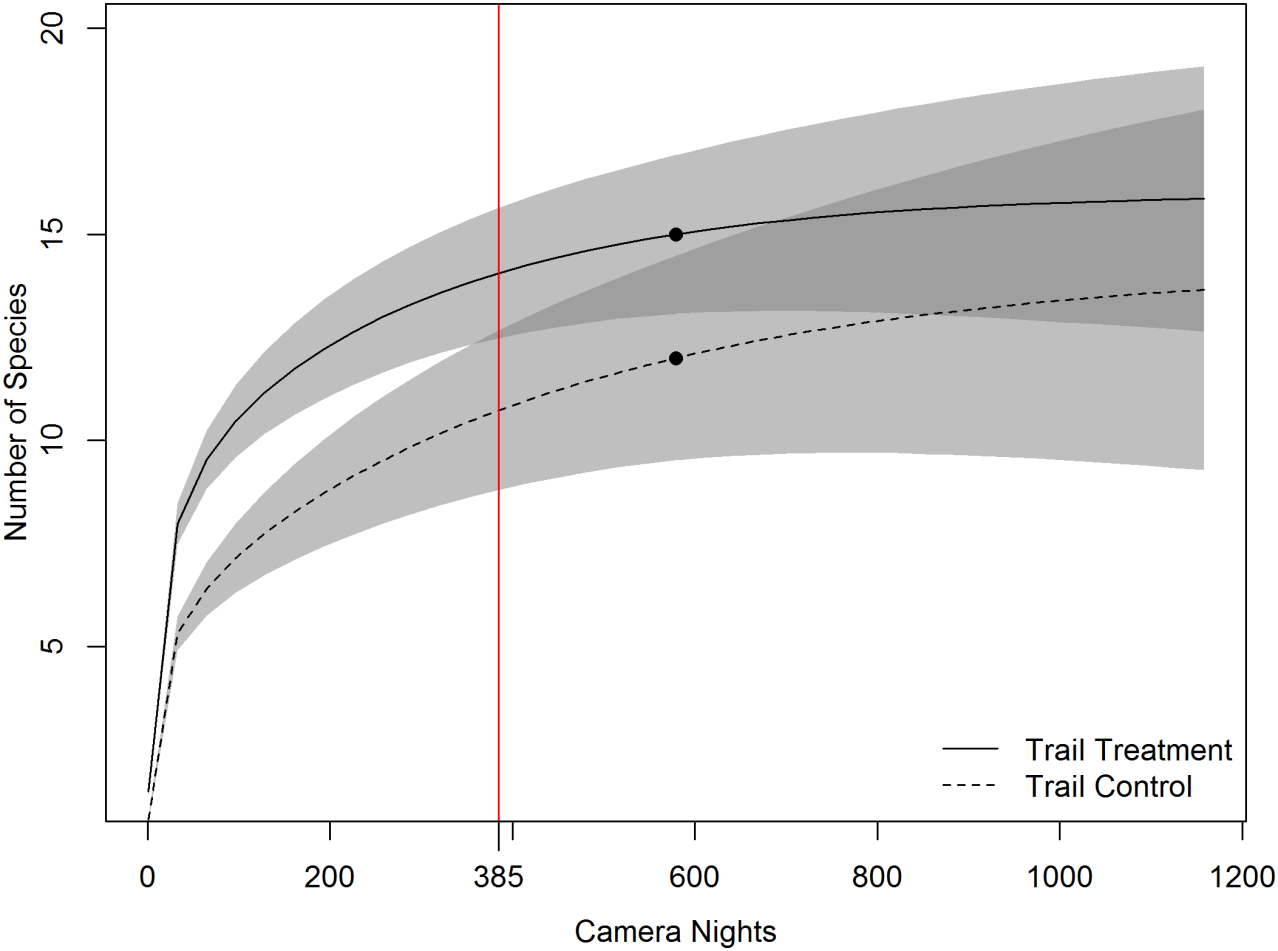
Sample-based species accumulation curve from 24 paired cameras, run for an average of 25.6 camera nights, with one camera in each pair oriented toward a trail feature, and the other at a nearby (mean = 23.5 m) random location. Shaded areas represent the 95% confidence intervals, with darker shaded areas representing confidence interval overlap between the two scenarios. Black dots indicate the actual sampling effort of this study. The vertical line with label indicates the point at which the confidence intervals of the feature and control groups overlap.

Species accumulation curves indicated that cameras with log features in view accumulated unique species more quickly than at paired control cameras (Fig 1). However, confidence intervals for richness estimates began to overlap after approximately 659 camera nights. Similarly, for our trail experiment, unique species were accumulated more quickly at cameras placed on game trails, compared with nearby control cameras, yet here richness values began to overlap, based on confidence intervals, after approximately 385 camera nights of effort (Fig 2).

### Occupancy modeling

Sample sizes for occupancy models were 29 paired stations for the log experiment (one of the 30 sampled log stations was removed due to errors in photo date assignment in the camera metadata that made occasion assignments impossible) and 24 for the trail experiment. Setting a camera trap on a game trail, compared to a random location, had a significant effect on detection probability for five of the seven species for which models could be constructed (Table 3). Detection probability was higher on game trails than control cameras for *O*. *virginianus*, *D*. *virginiana*, *T*. *striatus*, *S*. *carolinensis* and *S*. *floridanus*, and there was no influence of camera placement on detection probability for *P*. *lotor* or *U*. *americanus*. The effect of trail was particularly dramatic for *O*. *virginianus*, and this was the only species for which trail quality influenced detection as well, with trails of higher quality resulting in higher detectability (cumulative AICc weight 0.61; S1 Table). Increases in detection probability for these species ranged from 11–33% at median covariate values in the summer season, with Reconyx cameras (Table 4).

**Table 3:** Model results showing the effect of setting a camera on a feature (game trail or log) based on paired comparisons with nearby random locations.

Species	Trail Feature (*ΔAICc*)	Log Feature (*ΔAICc*)
*Odocoileus virginianus*	**+** (20.44)	- (37.77)^§^
*Didelphis virginiana*	**+** (8.09)	- (2.17)
Tamias striatus*	**+** (7.56)	*NA*
*Sciurus carolinensis*	**+** (2.28)^☩^	**+** (49.04)^¶^
Sylvilagus floridanus*	**+** (1.95)	*NA*
*Procyon lotor*	No effect (2.21)	**+** (3.82)
*Ursus americanus*	No effect (3.66)	No effect (4.11)
Mouse (*Peromyscus* sp.)^✝^	*NA*	**+** (37.78)
*Sciurus niger*^✝^	*NA*	**+** (23.24)
*Urocyon cinereoargenteus*	*NA*	No effect (3.69)

The data columns show the effect of feature presence on detection; the direction of this difference (positive [+] or negative [–]), and the *ΔAICc* value between the global detection model (all covariates) with and without feature type. An NA value indicates that there were insufficient detections for a given species in the camera pairs for a certain feature to allow investigation. All detection models were run with the top model for local occupancy (*θ*) of the species. The null model for theta (*θ* (.)) was the top model for almost all species; three exceptions are noted in the table.

* Trail data only sufficient to support basic models. Covariates on *θ* and *p* not investigated.

^✝^ Log data only sufficient to support basic models. Covariates on *θ* and *p* not investigated.

^§^The model used for calculating detection differences was *ψ*(.) *θ* (*CovAll*)

^☩^The model used for calculating detection differences was *ψ*(.) *θ* (*CovAll*)

^¶^The model used for calculating detection differences was *ψ*(.) *θ* (*global*)

**Table 4:** Percent increase or reduction in detection probability due to camera placement (trail or log).

	% Change in Detection Probability	
Species	Trail vs. Random	Log vs. Random
*Odocoileus virginianus*	+24.0%	-24.2%
*Procyon lotor*	no effect	+11.5%
*Ursus americanus*	no effect	no effect
*Sciurus carolinensis*	+17.3%	+27.4%
*Didelphis virginiana*	+11.7%	-9.3%
*Urocyon cinereoargenteus*	n/a	no effect
*Sciurus niger*	n/a	+24.9%*
*Sylvilagus floridanus*	+24.6%*	n/a
Mouse (*Peromyscus* sp.)	n/a	+38.2%*
*Tamias striatus*	+33.2%*	n/a

Detection probability was estimated based on the best model for *θ*, and the global (fully parameterized) model for a placement-specific *p*. Only values for species where camera placement was an important factor are shown. Median values for covariates on *p*, in the Summer, with a Reconyx camera were used to estimate *p* for feature vs. control setups. Refer to Tables 2 and 3 for model details. Those cases where data was not sufficient for modeling are indicated with an “n/a”.

* sample sizes did not allow for testing of all covariates. Effect of camera placement was tested in isolation, with no additional covariates.

Based on cumulative AICc model weight across balanced model sets, additional microhabitat variables also influenced detectability of certain species in the trail experiment. Detectability was higher at stations with lower understory stem densities for both *O*. *virginianus* and *S*. *carolinensis* (S1 Table). For *S*. *carolinensis* this same pattern held for low vegetative cover as well (S1 Table). Season influenced detectability for three species, with detectability higher in the summer season for *U*. *americanus* and *S*. *carolinensis*, and lower in the summer season for *P*. *lotor* (S1 Table), yet only three pairings occurred in the Fall for trail experiments. Camera type was generally not important for detectability with the exception of *P*. *lotor*, where Reconyx cameras had lower detectability than Spypoint (S1 Table).

Setting a camera trap facing a log had a strong influence on detectability for six of the eight species which could be modeled, and both positive (*S*. *carolinensis*, *S*. *niger*, Mouse, *P*. *lotor*) and negative (*O*. *virginianus*, *D*. *virginiana*) effects were demonstrated (Table 3). The impact of the log placement on detectability was most pronounced for the three rodents investigated, where in all cases detection probability was substantially higher (24.9–38.2%) at log cameras (Table 4). Log diameter was not an important covariate for detection probability at log-based cameras for any species.

As with the trail stations, additional microhabitat variables were important in determining detection probabilities at log stations. The amount of low vegetative cover had a strong negative influence on detection probability of *S*. *carolinensis*, which was opposite to that of the trail experiment where increasing low vegetative cover was associated with an increase in detection probability (S2 Table). Greater amounts of low vegetative cover also increased the detection probability of *U*. *cinereoargenteus* (S2 Table). Understory stem density had a strong positive effect on detection probability for both *P*. *lotor* and *S*. *carolinensis*, but strong negative effects on detection for *O*. *virginianus* and *U*. *cinereoargenteus* (S2 Table). *O*. *virginianus*, *P*. *lotor*, and *U*. *cinereoargenteus* detection probabilities were all higher in the Summer season compared with Fall. Camera type in log experiments was an important influence on detection probabilities for only two species (*O*. *virginianus* and *U*. *cinereoargenteus*) and in both cases detection probability was higher at pairings with Reconyx cameras (S2 Table).

Best models for *θ*(local occupancy) largely included none of our four possible habitat covariates, with a few exceptions. At trail experiment stations, local occupancy of *S*. *carolinensis* was higher at stations where overall amount of vegetative cover (CovAll) was higher. At log experiment stations, this pattern also held, with *S*. *carolinensis* local occupancy higher at locations where overall vegetative cover was higher (CovAll). However, for the same species, increases in overstem density (OverStem), understory stem density (UnderStem) and low vegetative cover (LowCov) all reduced the probability of local occupancy. Also at log experiment stations, *O*. *virginianus* local occupancy increased with decreasing overall vegetative cover.

## Discussion

Placement of remote cameras impacted the rate that animals were detected by camera traps, the total number of species detected, and detection probability across a suite of species. Biases identified were dramatic in some cases, with detection probability increases up to 38% and up to a 9.7 times increase in capture rate when cameras were set on certain features. Our results clearly indicate that additional small-scale habitat features besides game trails can have impacts on the ability to document unique species and the probability of species detection when camera traps are employed.

### Species richness

Most of the research concerning camera trap detection patterns and biases has focused on the impact of small roads and large human-use trails on the detectability of carnivores and their prey (e.g. [15, 16] [10, 17]), yet few have investigated potential biases when broad species richness is the information of interest (but see [30]). Our data indicate that relatively small trails unused by humans as well as other small features like downed logs can influence the total number of species photographed and the rate at which unique species are accumulated. Cameras set on log features resulted in the highest number of species (17) of all four camera groups, and showed a larger discrepancy between feature and control cameras than did the trail experiment. In terms of estimates of species richness, more than 650 camera nights are estimated to be necessary to remove this bias, which is a relatively large amount of effort for camera trap studies (e.g. 20 stations run for 30 days), and differences in richness values were most pronounced at low amounts of effort, as reported elsewhere where bias of small trails was investigated in a large community of 41 species of mammals in Tanzania [30]. Based on more than 6 years of camera trapping efforts on this property, and the known terrestrial mammals species that occur in our study location, there are no species missing from our list of 18 wild photographed species that would otherwise be expected to be captured with terrestrial, forest-based camera traps.

### Capture rates and relative abundance

Camera trapping studies that focus on multiple species typically report capture rates of photographed species, and this is often referred to as relative abundance or as a relative abundance index [9]. Yet, as noted previously [10] interpretation of this index is challenging as the potential biases affecting detection across species, and within species across study areas, are ignored. Although we did not have information on true abundance for our study species, we showed clear impacts of the presence of logs, and the presence and quality of trails within the camera view on capture rates, in a design that controlled for the impact of abundance. For relatively uncommon or low density species, these biases can result in a total lack of captures, thereby strongly influencing not only relative abundance, but richness estimates as well. Across the study, randomly placed (control) cameras did not photograph a single individual of southern flying squirrel (*G*. *volans*), least weasel (*M*. *nivalis*) or long-tailed weasel (*M*. *frenata*). Anecdotal observations from 687 deployments across four eastern US states and more than 14,000 camera nights support this finding, where the only detections of long-tailed weasels, least weasels, and southern flying squirrels were on opportunistically present log features [29]. These three species’ particular behavior and size combine to make them difficult to document on camera traps, regardless of their local densities. Although we recorded only a few events for these species, there is strong evidence that cameras focused on log features will increase photographic captures of these species in the eastern US. Rare or hard to detect species in other areas likely have features that are important to them; focusing on these features will increase detection rates and documenting the presence of these habitat features will be essential for accurately modeling detection of these species in community studies.

Biases in capture rates due to trails and logs were however apparent in even the most common species. Three of the five most commonly detected animals showed significant differences in capture rate between random cameras and cameras set on one of the feature types (Table 2), with increases in capture rates as high as 9.7 times. These differences could lead researchers to erroneous conclusions when comparing different areas or time periods if the relevant habitat features are not controlled for or otherwise accounted for in analytical approaches. The dangers of making conclusions based solely on capture rates, particularly across study areas, or where camera placements vary, have been demonstrated previously (e.g. [8]), and our data here support recommendations against this practice. Further, it seems the reluctance of many researchers to not place cameras in truly random locations for fear of large reductions in detection events is strongly supported. In this study randomly-placed cameras showed a 15.2% and 46.2% reduction in total detection events for log and trail pairs, respectively, with impacts of placement varying widely across different species.

### Detection probability

When investigated in an occupancy modeling framework, clear impacts of game trails and logs on the detection probabilities of a range of species were apparent. Positioning a camera trap on a game trail resulted in substantially higher detection probabilities for five of the seven species investigated compared to nearby random locations. While this was expected for *O*. *virginianus*, which were likely the creators of nearly all the game trails used in this study and which were detected more on larger game trails, other patterns were more surprising. While it is feasible to imagine that *D*. *virginiana* may utilize trails for travel to some degree, the remaining three species found to be more detectable on trails are smaller rodents and lagomorphs, all of which are unlikely to utilize trails for travel. It is therefore likely that the higher detectability on trail features may relate to higher visibility of these species in areas where the vegetation may otherwise make it challenging for cameras to detect small species. This is further supported by the fact that at trail stations, *S*. *carolinensis* detection was substantially reduced when low vegetative cover and understory stem densities were high. While detections in this study were not frequent enough to address this pattern in carnivores, we showed that even small game trails can be important features for species detection rates for a wide range of body sizes and taxa groups, from *T*. *striatus* to *O*. *virginianus*. While there is substantial research documenting the influence of trails and old roads on detection of carnivores [10, 14–17], and in some cases their prey [15, 18, 19], we are not aware of previous research showing these same patterns in small- and medium-sized mammals.

The size of trails or roads and other trail characteristics are typically not reported in camera trapping studies nor are they commonly incorporated into models for detection probability in an occupancy modeling framework. In our system where only game trails (as opposed to roads or human use trails) were considered, we show that *O*. *virginianus* were more easily detected on larger, more well-worn game trails. It should be noted that in the eastern US deer do not share these trails with any large predators. In Belize, researchers found that on large human-use trails, jaguar, puma and ocelot were photographed more often as trail width increased, and red brocket deer showed the opposite pattern [20], indicating differential response of predators and prey to different trail sizes. We recommend that camera studies using trails of any type record trail characteristics at camera locations and either use these factors in models explicitly estimating detection probability, or somehow control for potential variation in trail characteristics in the study design phase.

The effect of camera placement on detection probability was not limited to trails. Positioning a camera trap in view of a log, a heretofore ignored feature, resulted in substantially higher detection probabilities for four of eight species investigated. The impact on detection was dramatic for the three rodent species in this group, indicating that the presence of microhabitat features like this must be accounted for in camera placement for small mammals. As expected for a species primarily using travel pathways to move through the forest, *O*. *virginianus* detectability was lower at log cameras than at random locations. Interestingly, while increases in understory stem density, low vegetative cover, and overstory stem density reduced the probability of local occupancy of squirrels, these factors increased the detection of the species at log pairings, suggesting that perhaps log features are used more readily in densely vegetated habitats. This potential interaction between surrounding habitat and the importance of microhabitat features for detection probability is likely to occur for other features as well, and this possibility should be strongly considered when researchers collect field data and build covariate sets for model-based investigations. That is, the extent to which important microhabitat features influence detection rates may depend at least in part on other local habitat characteristics, and these should not be ignored.

Ours is the first study to investigate the influence of camera placement on detection of any bear species. Placement strategy had no influence on detectability of *U*. *americanus* and indeed none of our small-scale habitat covariates influenced either local occupancy or detectability for this species. It is likely that the omnivorous diet of this species renders extended travel along predetermined routes like game trails an inefficient foraging strategy.

Very few previous studies investigating factors influencing species detection on camera traps have used a truly controlled design with paired cameras as we have here [21, 22, 30], and only one to our knowledge has had more than 10 stations [30]. This paired design is essential for the control of other potential habitat characteristics that may also influence detectability, which we show can play a large role (e.g. stem densities and vegetative cover). Further, the use of the multi-method occupancy modeling approach explicitly accounts for the lack of independence between the two cameras resulting from their close proximity. We therefore strongly encourage other studies investigating detection bias to employ this design and analysis to properly incorporate the potential influence of spatial and temporal heterogeneity.

## Conclusions

Researchers have readily acknowledged that the detectability of animals on camera traps is impacted by behavioral (e.g. arboreality [38]), ecological [39], and biological (e.g. size [11]) variation across species. However, the role of more subtle factors on detection such as small scale local habitat features, within and across species, has been unclear. We show that small scale features associated with camera placement, including some that have been widely ignored in camera placement design and analysis (e.g. presence of logs, trail quality), may have strong impacts on detection in certain species. The resulting bias in detection will directly bias estimates of system state parameters like occupancy probability and therefore future camera study design may need to incorporate additional controls or model covariates to ensure robust estimates.

As always, study design should reflect the study objectives. Given the numerous potential factors influencing detection within and across species, including those heretofore not investigated, it seems clear that studies attempting to document richness and diversity should similarly maximize diversity in camera setups as much as possible. When faced with obvious bias in detection across species due to roads and trails, some researchers have recommended that camera locations include both road/trail features and more randomized locations, in unspecified proportions [15, 21, 22]. Yet for different species this will have unknown effects on detection and spatial and temporal bias, and there is no defensible method for selecting how to spatially and proportionally distribute random and targeted locations. We therefore agree with Cusack et al. [30] and Wearn et al. [16] in recommending random placements of camera traps for these studies. Although this study confirms results of previous research showing random locations will result in lower capture rates, we also show that bias in richness estimates can be overcome with large samples. More importantly, this approach should result in sampling of game trails, log features, and other microhabitats in proportion to their occurrence in the environment. Studies in this context that set cameras on game trails, roads, or a mix of trails and random locations will likely suffer from biased detection across species and make it challenging to interpret relative abundance derived from capture rates.

Those studies aiming to maximize capture of a single species (e.g. single-species occupancy and spatially-explicit capture-recapture approaches) by targeting specific habitat features should record characteristics associated with that feature (e.g. trail or road size), to model their potential effect on detection probability, particularly when spatial patterns across a landscape are of interest. The presence and characteristics of small-scale habitat features should be regularly recorded along with standard measures such as camera height, make/model and settings. Even these factors directly associated with cameras are often unreported [9, 40, 41], indicating that there is widespread lack of recognition of the importance of accounting for factors influencing detectability in study design.

Camera traps are a powerful tool for surveying mammal communities, and the more we know about the factors and mechanisms that influence detectability, the more accurately we can model abundance, diversity, and species interactions. The full continuum of factors influencing detectability in mammal communities is still not well understood, and the more understanding that we have of these factors, the more fully we can utilize camera traps to answer questions of scientific and conservation concern.

## Supporting information

S1 Table The influence of a trail feature on detection probability and cumulative AICc weights of habitat covariates. First column of data indicates whether camera placement on a game trail influenced detection probability of the given species, and in what direction (+ or -). The difference in AICc between the models with and without camera placement (*ΔAICc*) is also shown. Remaining columns show cumulative AICc weights across a balanced set of either 16 or 32 possible models (depending on whether placement was included) for five tested covariates on detection probability for 24 samples of trail pairings at 21 different grid locations across two years. Models were compared while holding *ψ* constant, and using the best model for *θ*(Four possible covariates: Low vegetative cover, Overall vegetative cover, Understory stem density, Overstory stem density). The sign (+ or -) of the relationship between detection probability (*p*) and the covariate is indicated in parentheses after cumulative weight values. Trail quality (coded “1–3”, with 1 highest quality) was tested as an interaction effect with camera placement, and is only reported for feature cameras (i.e. on trail). For the Season covariate, Summer was coded “1” with Fall coded “0”. For CamType, Reconyx cameras were coded “1” and Spypoint “0”.
(DOCX)

S2 Table The influence of a log feature on detection probability and cumulative AICc weights of habitat covariates. First column of data indicates whether camera placement with a log in view influenced detection probability of the given species, and in what direction (+ or -). The difference in AICc between the models with and without camera placement (*ΔAICc*) is also shown. Remaining columns show cumulative AICc weights across a balanced set of either 16 or 32 possible models (depending on whether camera placement was important) for five tested covariates on detection probability for 29 samples of log pairings at 23 different grid locations across two years. Models were compared while holding *ψ* constant, and using the best model for *θ*(Four possible covariates: Low vegetative cover, Overall vegetative cover, Understory stem density, Overstory stem density). The sign (+ or -) of the relationship between detection probability (*p*) and the covariate is indicated in parentheses after cumulative weight values. Log diameter effect was tested as an interaction effect with camera placement, and is only reported for feature cameras (i.e. log in view). For the Season covariate, Summer is coded “1” with Fall coded “0”. For CamType, Reconyx camera was coded “1” and Spypoint “0”.
(DOCX)
