# Supplementary material for: Camera trap placement and the potential for bias due to trails and other features
Source: PLoS One. 2017 Oct 18;12(10):e0186679. doi: 10.1371/journal.pone.0186679 (PMC5646845; doi:10.1371/journal.pone.0186679)
Supplement: S1 Table — First column of data indicates whether camera placement on a game trail influenced detection probability of the given species, and in what direction (+ or -). The difference in AICc between the models with and without camera placement (ΔAICc) is also shown. Remaining columns show cumulative AICc weights across a balanced set of either 16 or 32 possible models (depending on whether placement was included) for five tested covariates on detection probability for 24 samples of trail pairings at 21 different grid locations across two years. Models were compared while holding ψ constant, and using the best model for θ(Four possible covariates: Low vegetative cover, Overall vegetative cover, Understory stem density, Overstory stem density). The sign (+ or -) of the relationship between detection probability (p) and the covariate is indicated in parentheses after cumulative weight values. Trail quality (coded “1–3”, with 1 highest quality) was tested as an interaction effect with camera placement, and is only reported for feature cameras (i.e. on trail). For the Season covariate, Summer was coded “1” with Fall coded “0”. For CamType, Reconyx cameras were coded “1” and Spypoint “0”. (DOCX) [file pone.0186679.s001.docx]

**S1 Table. The influence of a trail feature on detection probability and cumulative AICc weights of habitat covariates.**

| Species | Trail $(\Delta AICc)$ | p  (CovLow) | p  (UndStD) | p  (Season)^b^ | p  (CamType) | p  (TrailQ) |
| --- | --- | --- | --- | --- | --- | --- |
| *Odocoileus virginianus*  $\psi(.) \theta(.)$ | Yes +  (20.44) | 0.172 (+) | 0.950 (-) | 0.237 (+) | 0.111 (-) | 0.607 (+) |
| *Didelphis virginiana*  $\psi(.) \theta(.)$ | Yes +  (8.09) | 0.172 (-) | 0.156 (-) | 0.185 (-) | 0.179 (-) | 0.045(+) |
| *Tamias striatus^a^*  $\psi(.) \theta(.)$ | Yes +  (7.56) | - | - | - | - | - |
| *Sciurus carolinensis*  $\psi(.) \theta(CovAll)$ | Yes +  (2.28) | 0.939 (-) | 1.0 (-) | 0.921 (+) | 0.132 (-) | 0.599 (-) |
| *Sylvilagus floridanus^a^*  $\psi(.) \theta(.)$ | Yes +  (1.95) | - | - | - | - | - |
| *Procyon lotor*  $\psi(.) \theta(.)$ | No  (2.21) | 0.179 (+) | 0.146 (-) | 0.984 (-) | 0.818 (-) | n/a |
| *Ursus americanus* $\psi(.) \theta(.)$ | No  (3.66) | 0.174 (+) | 0.167 (-) | 0.715 (+) | 0.253 (+) | n/a |

First column of data indicates whether camera setup on a game trail influenced detection probability of the given species, and in what direction (+ or -). The difference in AICc between the models with and without setup method ($\Delta AICc)$is also shown. Remaining columns show cumulative AICc weights across a balanced set of either 16 or 32 possible models (depending on whether setup was included) for 5 tested covariates on detection probability for 24 samples of trail pairings at 21 different grid locations across two years. Models were compared while holding $\psi$ (site occupancy) constant, and using an optimal model on $\theta$(Four possible covariates: Low vegetative cover, Overall vegetative cover, Understory stem density, Overstory stem density). The sign (+ or -) of the relationship between detection probability (*p*) and the covariate is indicated in parentheses after cumulative weight values. Trail quality (coded “1-3”, with 1 highest quality) was tested as an interaction effect with camera setup, and is only reported for treatment cameras (i.e. on trail). For the Season covariate, Summer was coded “1” with Fall coded “0”. For CamType, Reconyx cameras were coded “1” and Spypoint “0”.

^a^Data only sufficient to support basic models. Covariates on $\theta$ and *p* not investigated.

^b^Only 3 of the 24 trail pair deployments occurred in Fall vs. Summer, so these results should be viewed with caution.
