# Supplementary material for: Camera trap placement and the potential for bias due to trails and other features
Source: PLoS One. 2017 Oct 18;12(10):e0186679. doi: 10.1371/journal.pone.0186679 (PMC5646845; doi:10.1371/journal.pone.0186679)
Supplement: S2 Table — First column of data indicates whether camera placement with a log in view influenced detection probability of the given species, and in what direction (+ or -). The difference in AICc between the models with and without camera placement (ΔAICc) is also shown. Remaining columns show cumulative AICc weights across a balanced set of either 16 or 32 possible models (depending on whether camera placement was important) for five tested covariates on detection probability for 29 samples of log pairings at 23 different grid locations across two years. Models were compared while holding ψ constant, and using the best model for θ(Four possible covariates: Low vegetative cover, Overall vegetative cover, Understory stem density, Overstory stem density). The sign (+ or -) of the relationship between detection probability (p) and the covariate is indicated in parentheses after cumulative weight values. Log diameter effect was tested as an interaction effect with camera placement, and is only reported for feature cameras (i.e. log in view). For the Season covariate, Summer is coded “1” with Fall coded “0”. For CamType, Reconyx camera was coded “1” and Spypoint “0”. (DOCX) [file pone.0186679.s002.docx]

**S2 Table.** **The influence of a log feature on detection probability and cumulative AICc weights of habitat covariates.**

| Species | Log View  ($\Delta AICc$) | p  (CovLow) | p  (UndStD) | p  (Season) | p  (CamType) | p  (LogD) |
| --- | --- | --- | --- | --- | --- | --- |
| *Sciurus carolinensis*  $\psi(.) \theta(general)$ | Yes +  (49.04) | 0.999 (+) | 1.00 (+) | 0.239 (-) | 0.064 (+) | 0.005 (-) |
| *Peromyscus sp.*^a^  $\psi(.) \theta(.)$ | Yes +  (37.78) | - | - | - | - | - |
| *Odocoileus virginianus*$\psi(.) \theta(CovAll)$ | Yes -  (37.77) | 0.123 (-) | 0.708 (-) | 0.800 (+) | 0.991 (+) | 0.052 (+) |
| *Sciurus niger*^a^  $\psi(.) \theta(.)$ | Yes +  (23.24) | - | - | - | - | - |
| *Procyon lotor*  $\psi(.) \theta(.)$ | Yes +  (3.82) | 0.458 (+) | 1.00 (+) | 0.688(+) | 0.225 (-) | 0.050 (+) |
| *Didelphis virginiana*  $\psi(.) \theta(.)$ | Yes -  (2.17) | 0.177 (+) | 0.176 (+) | 0.275 (+) | 0.328 (+) | 0.048 (+) |
| *Ursus americanus*  $\psi(.) \theta(.)$ | No  (4.11) | 0.368 (+) | 0.190 (+) | 0.207(+) | 0.320 (-) | n/a |
| *Urocyon cinereoargenteus*$\psi(.) \theta(.)$ | No  (3.69) | 0.606 (+) | 0.676 (-) | 0.978(+) | 0.919 (+) | n/a |

First column of data indicates whether camera setup with a log in view influenced detection probability of the given species, and in what direction (+ or -). The difference in AICc between the models with and without setup method ($\Delta AICc)$is also shown. Remaining columns show cumulative AICc weights across a balanced set of either 16 or 32 possible models (depending on whether camera setup was important) for 5 tested covariates on detection probability for 29 samples of log pairings at 23 different grid locations across two years. Models were compared while holding $\psi$ (site occupancy) constant, and using an optimal model on $\theta$(Four possible covariates: Low vegetative cover, Overall vegetative cover, Understory stem density, Overstory stem density). The sign (+ or -) of the relationship between detection probability (*p*) and the covariate is indicated in parentheses after cumulative weight values. Log diameter effect was tested as an interaction effect with camera setup, and is only reported for treatment cameras (i.e. log in view). For the Season covariate, Summer is coded “1” with Fall coded “0”. For CamType, Reconyx camera was coded “1” and Spypoint “0”.

^a^ data only sufficient to support basic models. Covariates on $\theta$ and *p* not investigated.
